# Access to care for patients with chronic pain receiving prescription opioids, cannabis, or other treatments

**DOI:** 10.1093/haschl/qxae086

**Published:** 2024-06-12

**Authors:** Mark C Bicket, Elizabeth M Stone, Kayla Tormohlen, Reekarl Pierre, Emma E McGinty

**Affiliations:** Department of Anesthesiology, University of Michigan, Ann Arbor, MI 48108, United States; Opioid Prescribing Engagement Network, Institute for Healthcare Policy and Innovation, University of Michigan School of Public Health, University of Michigan, Ann Arbor, MI 48109, United States; Department of Psychiatry, Rutgers Robert Wood Johnson Medical School, New Brunswick, NJ 08901, United States; Center for Health Services Research, Rutgers Institute for Health, Health Care Policy, and Aging Research, New Brunswick, NJ 08901, United States; Division of Healthcare Policy and Economics, Weill Cornell Medical College, New York, NY 10065, United States; Division of Healthcare Policy and Economics, Weill Cornell Medical College, New York, NY 10065, United States; Division of Healthcare Policy and Economics, Weill Cornell Medical College, New York, NY 10065, United States

**Keywords:** access to care, prescription opioids, opioid analgesics, cannabis, medical marijuana, survey, primary care, chronic pain

## Abstract

Changes in chronic noncancer pain treatment have led to decreases in prescribing of opioids and increases in the availability of medical cannabis, despite its federal prohibition. Patients may face barriers to establishing new care with a physician based on use of these treatments. We compared physician willingness to accept patients based on prescription opioid, cannabis, or other pain treatment use. This study of 36 states and Washington, DC, with active medical cannabis programs surveyed physicians who treat patients with chronic noncancer pain between July 13 and August 4, 2023. Of 1000 physician respondents (34.5% female, 63.2% White, 78.1% primary care), 852 reported accepting new patients with chronic pain. Among those accepting new patients with chronic pain, more physicians reported that they would not accept new patients taking prescription opioids (20.0%) or cannabis (12.7%) than those taking nonopioid prescription analgesics (0.1%). In contrast, 68.1% reported willingness to accept new patients using prescribed opioids on a daily basis. For cannabis, physicians were more likely to accept new patients accessing cannabis through medical programs (81.6%) than from other sources (60.2%). Access to care for persons with chronic noncancer pain appears to be the most restricted among those taking prescription opioids, although patients taking cannabis may also encounter reduced access.

## Teaser

How willing are physicians to accept new patients with chronic noncancer pain based on whether they use prescription opioids, cannabis, or other treatments for pain management? In this national survey of physicians who treat patients with chronic noncancer pain, more physicians reported that they would not accept new patients who manage their chronic pain by taking prescription opioids (20.0%) than those who take cannabis (12.7%). In contrast, physicians rarely reported that they would not accept new patients who manage pain with nonopioid prescription analgesics (0.1%). Findings in this study indicate that access to chronic noncancer pain care may be the most restricted for patients taking prescription opioids, although patients taking cannabis may also encounter reduced access, which could inadvertently encourage these patients to seek nonmedical treatments for chronic pain.

## Introduction

The treatment landscape for chronic noncancer pain, which impacts more than 1 in 5 Americans,^1^ has undergone significant shifts over the last decade. Treatment with prescription opioids has broadly declined in response to changing clinical guidelines and other policies designed to curb opioid misuse,^2^ and the use of cannabis for chronic pain management has increased with state legalization of cannabis for medical conditions.^3^ Contemporary guidelines emphasize the use of nonopioid, non-cannabis options as first-line treatment for chronic noncancer pain.^4-6^ Anecdotally, there is stigma around prescription opioid use for chronic pain.^7^ As physicians move away from treating pain with opioids, they may be less willing to accept new patients using prescription opioids to manage pain. Physicians may also be uncomfortable managing patients using cannabis, given that its use is not guideline-concordant and remains prohibited under federal law.

Consequently, patients with chronic noncancer pain may face barriers to initiating treatment with a physician if they use prescription opioids or cannabis for pain management. While caring for patients with chronic pain has been cited as one of the most difficult issues encountered by physicians, no studies have examined how access varies based on patients' use of different types of pain treatments.^8^ To address these gaps, this investigation analyzed a national survey of US physicians treating patients with chronic noncancer pain in states with active medical cannabis programs in 2023. This study assessed physicians' willingness to accept new patients with chronic pain using prescription opioids, cannabis, and nonopioid prescription analgesics.

## Data and methods

In this cross-sectional web survey, we examined a national sample of physicians practicing in the 36 states and Washington, DC, with active medical cannabis programs in July–August 2023. Ipsos fielded the survey using the SurveyHealthcareGlobus physician survey panel. This opt-in panel includes approximately 800 000 US physicians (∼75% of active US physicians) recruited from the American Medical Association (AMA) Masterfile, hospital directories, and other verified medical directories of physicians. For this study, physicians with specialties that commonly treat chronic noncancer pain (family medicine, internal medical, general medicine, anesthesiology, neurology, physical medicine, and rehabilitation) were included in the survey sample if they reported spending 50% or more of their professional time caring for patients, if they cared for 100 or more patients in the past year, and if they cared for any patients with chronic noncancer pain in an outpatient clinical setting in the past year. A screening survey identified eligible physicians, who were then invited via email to participate in a survey on “chronic non-cancer pain management.” The survey was fielded from July 13, 2023, to August 4, 2023 (additional details in the eMethods). Respondents received an incentive between $20 and $30 for their participation in the survey.

We first asked physicians about their behaviors of whether they were accepting any new patients with chronic noncancer pain. Among physicians who responded “yes” to accepting new patients with chronic noncancer pain, we examined responses to the following questions about type of pain treatment: “Do you currently accept new patients with chronic noncancer pain who are managing their pain with [prescription opioids, cannabis, nonopioid prescription analgesics]?” Physicians who reported accepting new patients using opioids were asked about their preferences of whether they would accept patients who take prescribed opioids “on a daily basis” for pain. Physicians who reported accepting new patients using cannabis were asked whether they would accept “patients accessing cannabis through the state medical cannabis program” and/or “patients using cannabis obtained from sources other than the state medical cannabis program.” Survey questions only allowed for binary (yes/no) responses. We calculated the proportion responding “yes” to each of these items.

All analyses incorporated survey sampling weights to generate estimates representative of physicians in the 36 states and Washington, DC, included in the sample, with the AMA physician Masterfile data used as the sample weighting benchmark. The variables analyzed in this report did not have missing data. The Weill Cornell Medical College Institutional Review Board approved this study.

## Results

Of 1372 physicians identified as eligible, 1000 (73%) completed the full survey (median age [SD], 52 [11.3] years; 34.5% female; 35.9% non-White) (eFigure S1). Most identified as primary care physicians (78.1%), and nearly half of physicians (46.5%) treated a panel with a proportion of patients with chronic pain between 1% and 33% (eTable S1). Only 26.7% of physicians reported completion of their state's authorization process for formally recommending patients for use of cannabis through the state program.

**Figure 1.**
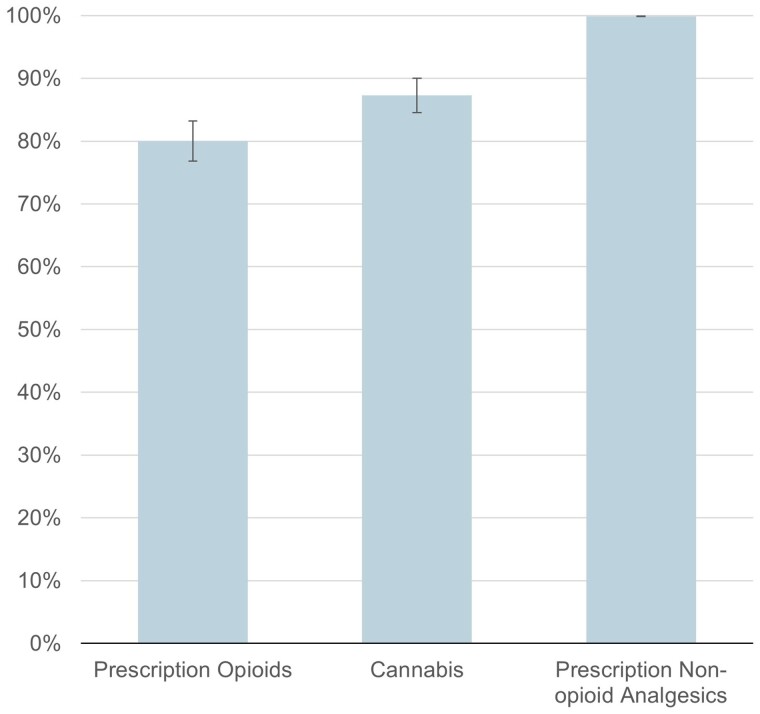
Proportion of physicians accepting new patients with chronic noncancer pain by type of existing chronic pain treatment. Measures are from a survey of physicians fielded by Ipsos using the SurveyHealthcareGlobus panel fielded from July 13, 2023, to August 4, 2023, among respondents willing to accept new patients with chronic noncancer pain (*n* = 852). Measures signify the proportion responding “yes” to the question: “Do you currently accept new patients with chronic noncancer pain who are managing their pain with [prescription opioids, cannabis, nonopioid prescription analgesics]?” Error bars show 95% CIs. Results account for sampling weights.

Overall, 82.8% of physicians reported currently accepting any new patients with chronic pain. Among this group, 20.0% (95% CI, 16.8%–23.2%) of physicians were not willing to accept new patients taking prescription opioids and 12.7% (95% CI, 9.9%–15.4%) were not willing to accept new patients taking cannabis. In contrast, 0.1% (95% CI 0.0%–0.2%) of physicians were unwilling to accept new patients taking nonopioid prescription analgesics (Figure 1). Physician characteristics did not differ among those who were not willing to accept patients taking prescription opioids when compared with those who were not willing to accept patients taking cannabis (eTable S2).

**Figure 2.**
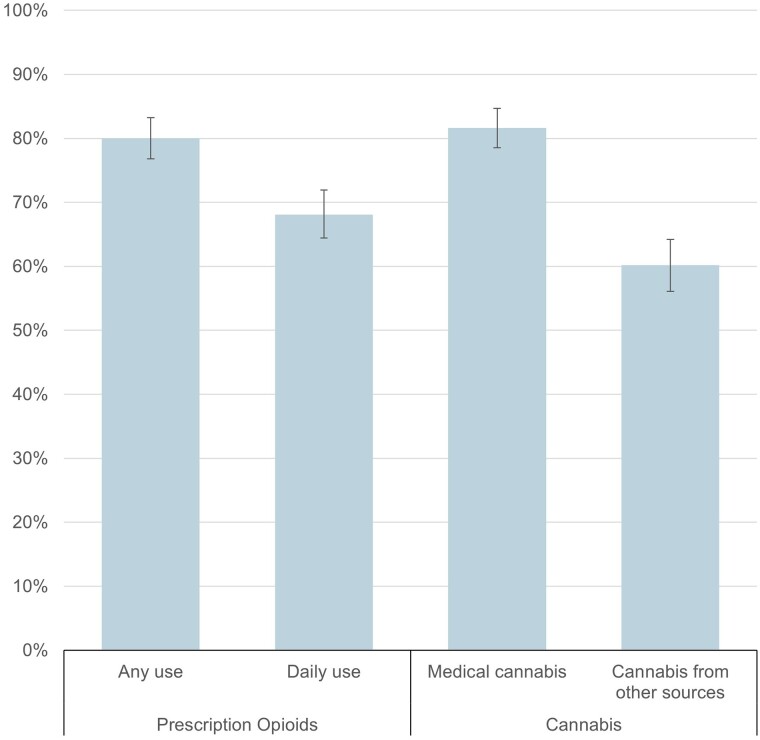
Proportion of physicians willing to accept new patients with chronic noncancer pain by frequency of prescription opioid use and source of cannabis for pain management. Measures are from a survey of physicians fielded by Ipsos using the SurveyHealthcareGlobus panel fielded from July 13, 2023, to August 4, 2023, among respondents willing to accept new patients with chronic noncancer pain (*n* = 852). Physicians were asked if they currently accept new patients with chronic noncancer pain who are managing their pain with prescription opioids and, among those who said yes, whether they accepted patients who take prescribed opioids “on a daily basis” to manage their pain. Physicians who reported accepting new patients using cannabis were asked whether they accepted “patients accessing cannabis through the state medical cannabis program” and/or “patients using cannabis obtained from sources other than the state medical cannabis program.” Error bars show 95% CIs. Results account for sampling weights.

The proportion of physicians willing to accept new patients with chronic pain varied based on characteristics of analgesic use (Figure 2). For prescription opioids, while 80.0% (95% CI, 76.8%–83.2%) reported willingness to accept patients using any prescription opioids, 68.1% (95% CI, 64.4%–71.9%) reported willingness to accept new patients taking prescription opioids on a daily basis. For cannabis, physicians were more likely to accept patients accessing cannabis through medical cannabis programs (81.6%; 95% CI, 78.5%–84.7%) than those using cannabis obtained from other sources (60.2%; 95% CI, 56.1%–64.2%).

## Discussion

Among physicians actively accepting new patients with chronic pain, 20.0% were unwilling to accept a new patient taking prescription opioids, while 12.7% were unwilling to accept a new patient taking cannabis. In contrast, few physicians (0.1%) were unwilling to accept a new patient taking nonopioid prescription analgesics. Physician acceptance of new patients was lower for patients using prescribed opioids on a daily basis and higher for those using cannabis obtained from medical programs compared with cannabis from other sources.

These findings build upon the small number of state-based studies that have uncovered reluctance among physicians to treat new patients taking prescription opioids.^9,10^ A phone-based audit survey of primary care clinics in Michigan found that 41% of 79 clinics that were contacted would not accept new patients receiving prescription opioids as a treatment for chronic pain.^10^ Study findings also suggest that people using medical cannabis for pain management may face access barriers. This lack of access could inadvertently encourage patients to seek nonmedical treatments for their chronic pain, given that relief of pain is the most commonly reported reason for misuse of controlled substances.^11^ In response to concerns about difficulty for patients to obtain care, some states have passed legislation that prohibits physicians from denying care to persons who take cannabis, such as California AB-1954.^12^

For both prescription opioids and medical cannabis, gaps exist regarding high-quality studies that critically examine their effectiveness as long-term treatments for chronic noncancer pain, which may contribute to uncertainty regarding their analgesic use.^3,13^ Physicians endorse a general lack of knowledge on the clinical risks as well as benefits of cannabis to manage chronic noncancer pain.^14^ For prescription opioids, evidence on their long-term effectiveness for chronic noncancer pain remains very limited, while the risk of harm appears to be dose dependent.^15^

Limitations of this analysis include its use of a convenience sample, although the physician panel used includes 75% of active physicians in the United States and analyses were weighted to representative benchmarks. Physicians' responses may be influenced by social desirability bias, a concern we worked to mitigate by use of an anonymous survey. While this analysis examines factors that correlate with some survey responses, examination of factors with all survey responses was not performed. Finally, the inclusion of binary responses to questions asking about actual practices regarding the acceptance of patients may not capture whether providers consider the use of these treatments in their patient acceptance decisions and instead inadvertently lead to reporting of preferences rather than actual practices.

In conclusion, these results indicate that access to care may be the most restricted for patients taking prescription opioids, although patients taking cannabis may also encounter reduced access.

## Supplementary Material

qxae086_Supplementary_Data

## Data Availability

The data will not be shared per the data use agreement with Ipsos, which only allows access by the study team.

## Access to data and data analysis

Dr. Stone had full access to all the data in the study, analyzed the data, and takes responsibility for the integrity of the data and the accuracy of the data analysis.
