# Supplementary material for: Access to care for patients with chronic pain receiving prescription opioids, cannabis, or other treatments
Source: Health Aff Sch. 2024 Jun 12;2(6):qxae086. doi: 10.1093/haschl/qxae086 (PMC11210295; doi:10.1093/haschl/qxae086)
Supplement: qxae086_Supplementary_Data [file qxae086_supplementary_data.zip › Supplement 20240528.docx]

**Supplement for**

Bicket MC, Stone EM, Tormohlen K, Pierre R, McGinty EE. Access to Care for Patients with Chronic Pain Receiving Prescription Opioids, Cannabis, or Other Treatments. Health Affairs Scholar 2024. https://doi.org/10.1093/haschl/qxae086

**eMethods.** Additional methods and list of questions from physician survey

**eTable 1.** Characteristics of Physicians

**eTable 2.**  Among physicians accepting new chronic pain patients, characteristics of physicians who are unwilling to accept new chronic pain patients managing their pain with prescription opioids or medical cannabis

**eFigure 1**. Flow diagram of physician survey

**eMethods.**

The target population was physicians providing ambulatory clinical care for chronic pain patients in states with medical cannabis programs. Based on federal districts and states with medical cannabis programs at the time of the survey, physicians from the District of Columbia and the following 36 states were included: Alaska, Alabama, Arkansas, Arizona, California, Colorado, Connecticut, District of Columbia, Delaware, Florida, Hawaii, Illinois, Louisiana, Massachusetts, Maryland, Maine, Michigan, Minnesota, Missouri, Montana, North Dakota, New Hampshire, New Jersey, New Mexico, Nevada, New York, Oregon, Ohio, Oklahoma, Pennsylvania, Rhode Island, South Dakota, Utah, Virginia, Vermont, Washington, West Virginia.

Pretesting of the questionnaire was performed on the first two days of survey administration. We excluded physicians who reported devoting less than 50% of clinical time to the management of a panel of patients, those evaluating or treating less than 100 patients in the past year, and those who had not evaluated or treated any patients with chronic pain in the outpatient clinical setting. For questions relevant to this analysis, respondents completed sections on treatment of chronic pain, treatment attitudes and practices, and respondent characteristics. The survey was administered online using the via Ipsos, which fielded the survey using the SurveyHealthcareGlobus physician survey panel. This opt-in panel includes ~800,000 U.S. physicians (~75% of active U.S. physicians) recruited from the American Medical Association (AMA) Masterfile, hospital directories, and other verified medical directories of physicians. For this study, physicians with specialties that commonly treat chronic noncancer pain (family medicine, internal medical, general medicine, anesthesiology, neurology, physical medicine and rehabilitation) were included in the survey Potential respondents were invited to participate in the survey using a secure link unique to the respondent. One additional reminder was provided, with expiration of the link to prevent multiple participation. A total 85,670 invitations were sent to physicians regarding participation in the survey. Among those invitations, 1,372 entered the screener.

Respondents were provided with additional descriptive text examples to clarify prescription opioids (“*Examples of prescription opioids include hydrocodone (Vicodin, Lortab); oxycodone (Percocet, Tylox, Percocet); hydromorphone (Dilaudid); morphine; codeine (Tylenol #3); tramadol (Ultram); meperidine (Demerol); tapentadol (Nucynta); fentanyl (Actiq)]*”) and non-opioid prescription analgesics (“*Examples of common nonopioid analgesics include prescription-strength acetaminophen/Tylenol, NSAIDs like prescription-strength ibuprofen, Gabapentin/Neurontin, pregabalin/Lyrica, Tricyclic antidepressants (TCAs) such as nortriptyline, amitriptyline, Serotonin-Norepinephrine reuptake inhibitors (SNRIs) such as duloxetine, and topical agents such as lidocaine, capsaicin.]*”).

Statistical analysis was performed using Stata 18.0.

**List of questions from physician survey**

**Section: Screening questions**

What is your date of birth?
(picker for year 1910-2015; picker for month January-December)

Please insert your zip code
(*Respondents from the states of Alaska, Alabama, Arkansas, Arizona, California, Colorado, Connecticut, District of Columbia, Delaware, Florida, Hawaii, Illinois, Louisiana, Massachusetts, Maryland, Maine, Michigan, Minnesota, Missouri, Montana, North Dakota, New Hampshire, New Jersey, New Mexico, Nevada, New York, Oregon, Ohio, Oklahoma, Pennsylvania, Rhode Island, South Dakota, Utah, Virginia, Vermont, Washington, West Virginia will continue. If other state, respondents will screen out)*

In the past year, what proportion of your clinical time has involved the management of acute or chronic conditions for a panel of patients?

1. Less than 50%

2. 50% or more

(Respondents answering 2 will continue and 1 will screen out)

In the past year, have you evaluated or treated at least 100 patients?

1. Yes

2. No

(Respondents answering 1 will continue and 2 will screen out)

In the past year, have you evaluated or treated any patients with chronic noncancer pain in an office or other outpatient clinical setting?

1. Yes

2. No

(Respondents answering 1 will continue and 2 will screen out)

Which of the following best describes your primary medical specialty?

a. Family Medicine

b. Internal Medicine

c. General Medicine

d. Anesthesiology

e. Neurology

f. Physical medicine and rehabilitation

g. Pain specialist

**Section: Treatment of Chronic Pain**

What proportion of your patient panel would you say has chronic noncancer pain, defined as pain that occurs on most or all days for three months or longer?
 Slider bar from 0-100%

Does your state have an authorization process requiring physicians to complete training, pass an examination, and/or register with the state in order to recommend or “certify” medical cannabis use for a patient?

a. Yes

b. No

c. I am not sure if my state has this type of authorization process

Have you completed this authorization process?

1. Yes

2. No

**Section: Treatment Attitudes and Practices**

Do you currently accept new patients

1. Yes
2. No

Do you currently accept new patients with chronic noncancer pain?

1. Yes
2. No

Do you currently accept new patients with chronic noncancer pain who are managing their pain with…
 a. Prescription opioids?
 b. Cannabis?
 c. Nonopioid prescription analgesics?

Responses:

1. Yes
2. No

You mentioned you currently accept new patients with chronic noncancer pain who are managing their pain with prescription opioids. Would you accept… (Select all that apply)
1. People who take prescribed opioids on a daily basis to manage their pain
2. People who take prescribed opioids less than daily to manage their pain

You mentioned you currently accept new [If Q17=2 show: would accept new] patients with chronic noncancer pain who are managing their pain with cannabis. Would you accept… (Select all that apply)
1. Patients accessing cannabis through the state medical cannabis program
2. Patients using cannabis obtained from sources other than the state medical cannabis program

**Section: Respondent characteristics**

What is your gender?

a. Man

b. Woman

c. Non-binary

d. Transgender

e. None of these describe me, and I’d like to consider additional options

f. Prefer not to say [Exclusive]

Ask if c,d, or e
Are any of these a closer description to your gender identity?

a. Trans man/Transgender man/FTM

b. Trans woman/Transgender woman/MTF

c. Genderqueer

d. Genderfluid

e. Gender variant

f. Questioning or unsure of gender identity

g. None of these describe me, and I want to specify [fill in blank]

h. Prefer not to say [Exclusive]

What is your race?

a. American Indian or Alaska Native

b. Asian or Asian American

c. Black or African American

d. Middle Eastern or North African

e. Native Hawai’ian or Pacific Islander

f. White or European

g. Other [specify]

h. Prefer not to say [Exclusive]

Are you of Hispanic, Latino, or Spanish origin?

a. Yes

b. No

c. Prefer not to say [Exclusive]

When did you graduate from medical school?

a. 2018 or later

b. 2013 to 2017

c. 2008 to 2012

d. 2003 to 2007

e. 1998 to 2002

f. 1997 or earlier

Which of the following best describe your current practice setting?

a. Solo practice

b. Group practice

c. Hospital setting (i.e., emergency department or outpatient department)

d. Community health center

e. Other

**eTable 1**. Characteristics of Physicians

| Characteristic | All Physicians (N=1000) | Physicians Accepting New Patients with Chronic Pain (N=852) |
| --- | --- | --- |
|  | N (weighted %) | N (weighted %) |
| Age, median (SD) | 52 (11.3) | 52 (11.3) |
| Gender | | |
| Man | 689 (63.0) | 610 (66.1) |
| Woman | 262 (34.5) | 206 (31.5) |
| Other | 7 (0.7) | 5 (0.7) |
| Prefer not to say | 42 (1.9) | 31 (1.7) |
| Race and Ethnicity | | |
| White | 592 (63.2) | 508 (62.8) |
| Asian | 230 (23.1) | 197 (23.1) |
| Black | 27 (6.3) | 21 (6.4) |
| Hispanic or Latino | 49 (6.5) | 39 (6.4) |
| Proportion of patient panel with chronic noncancer pain | | |
| Low (1% to 33%) | 428 (46.5) | 342 (44.5) |
| Medium (34% to 66%) | 310 (32.7) | 266 (32.5) |
| High (67% to 100%) | 262 (20.8) | 244 (23.0) |
| Completion of state cannabis authorization process | | |
| No | 363 (38.8) | 301 (37.0) |
| Yes | 295 (26.7) | 266 (28.8) |
| Other | 342 (34.5) | 285 (34.2) |
| Medical school graduation year | | |
| 2007 or earlier | 747 (77.6) | 626 (75.8) |
| 2008 or later | 253 (22.4) | 226 (24.2) |
| Primary medical specialty | | |
| Primary care | 641 (78.1) | 524 (76.4) |
| Pain specialists | 359 (21.9) | 328 (23.6) |
| Current practice setting | | |
| Solo or group practice | 751 (76.0) | 633 (74.6) |
| Hospital | 199 (18.6) | 176 (19.9) |
| Other (e.g., community health center) | 50 (5.4) | 43 (5.5) |

Notes: Results account for sampling weights. Other authorization process indicates state does not require authorization or unsure if state requires authorization. Other gender indicates Non-binary / Trans / Genderqueer / Genderfluid. Primary care specialties included family medicine, internal medicine, and general medicine. Pain specialists included anesthesiology, neurology, physical med/rehab, and pain specialist.

**eTable 2**. Among physicians accepting new chronic pain patients, characteristics of physicians who are unwilling to accept new chronic pain patients managing their pain with prescription opioids or medical cannabis

| Characteristic | Physicians Accepting New Patients with Chronic Pain | |
| --- | --- | --- |
|  | Taking Prescription Opioids | Taking Cannabis |
|  | N (weighted %) | N (weighted %) |
| N | 187 (20.0) | 108 (12.7) |
| Age, median (SD) | 54 (10.7) | 53 (11.6) |
| Gender | | |
| Man | 133 (70.0) | 72 (64.5) |
| Woman | 44 (27.6) | 32 (33.2) |
| Other | 1 (0.1) | 0 (0) |
| Prefer not to say | 9 (2.3) | 4 (2.3) |
| Race and Ethnicity | | |
| White | 113 (65.4) | 47 (44.7) |
| Asian | 40 (25.0) | 35 (37.5) |
| Black | 1 (2.0) | 2 (4.2) |
| Hispanic or Latino | 6 (2.6) | 2 (2.0) |
| Proportion of patient panel with chronic noncancer pain | | |
| Low (1% to 33%) | 85 (50.7) | 36 (32.7) |
| Medium (34% to 66%) | 57 (31.3) | 41 (44.6) |
| High (67% to 100%) | 45 (17.9) | 31 (22.7) |
| Completion of state cannabis authorization process | | |
| No | 64 (34.4) | 43 (38.6) |
| Yes | 59 (30.6) | 24 (23.2) |
| Other | 64 (35.0) | 41 (38.3) |
| Medical school graduation year | | |
| 2007 or earlier | 147 (79.9) | 86 (78.2) |
| 2008 or later | 40 (20.2) | 22 (21.8) |
| Primary medical specialty | | |
| Primary care | 114 (79.6) | 70 (82.0) |
| Pain specialists | 73 (20.5) | 38 (18.0) |
| Current practice setting | | |
| Solo or group practice | 151 (83.2) | 81 (74.2) |
| Hospital | 27 (11.2) | 20 (19.3) |
| Other (e.g., community health center) | 9 (5.6) | 7 (6.5) |

Notes: Results account for sampling weights. Other authorization process indicates state does not require authorization or unsure if state requires authorization. Other gender indicates Non-binary / Trans / Genderqueer / Genderfluid. Primary care specialties included family medicine, internal medicine, and general medicine. Pain specialists included anesthesiology, neurology, physical med/rehab, and pain specialist.

**eFigure 1**. Flow diagram of physician survey

**1,372** Physicians screened

**1,000** Physicians surveyed

**372** Excluded

**-** Did not spend 50% or more time in patient care

**-** Did not evaluate or treat at least 100 patients in the past year

**-** Did not evaluate or treat a patient with chronic pain in the outpatient setting

**148** Physicians not accepting new patients with chronic pain

**852** Physicians accepting new patients with chronic pain
